# Workplace Interventions to Prevent Disability from Both the Scientific and Practice Perspectives: A Comparison of Scientific Literature, Grey Literature and Stakeholder Observations

**DOI:** 10.1007/s10926-016-9664-z

**Published:** 2016-09-10

**Authors:** Kelly Williams-Whitt, Ute Bültmann, Benjamin Amick, Fehmidah Munir, Torill H. Tveito, Johannes R. Anema, the Hopkinton Conference Working Group on Workplace Disability Prevention, Benjamin C. Amick, Johannes R. Anema, Elyssa Besen, Peter Blanck, Cécile R. L. Boot, Ute Bültmann, Chetwyn C. H. Chan, George L. Delclos, Kerstin Ekberg, Mark G. Ehrhart, Jean-Baptiste Fassier, Michael Feuerstein, David Gimeno, Vicki L. Kristman, Steven J. Linton, Chris J. Main, Fehmidah Munir, Michael K. Nicholas, Glenn Pransky, William S. Shaw, Michael J. Sullivan, Lois E. Tetrick, Torill H. Tveito, Eira Viikari-Juntura, Kelly Williams-Whitt, Amanda E. Young

**Affiliations:** 1 University of Lethbridge, 4401 University Drive, Lethbridge, AB T1K 3M4 Canada; 2 University Medical Center Groningen, Community and Occupational Medicine, University of Groningen, Groningen, The Netherlands; 3 Robert Stempel College of Public Health and Social Work, Florida International University, Miami, FL USA; 4 Institute for Work and Health, Toronto, Canada; 5 School of Sport, Exercise and Health Sciences, Loughborough University, Loughborough, Leicestershire UK; 6 Uni Research Health, Bergen, Norway; 7 Department of Health Promotion, University College of Southeast Norway, Horten, Norway; 8 VU University Medical Center, Amsterdam, The Netherlands

**Keywords:** Workplace interventions, Disability prevention, Employer practices, Research priorities

## Abstract

*Purpose* The significant individual and societal burden of work disability could be reduced if supportive workplace strategies could be added to evidence-based clinical treatment and rehabilitation to improve return-to-work (RTW) and other disability outcomes. The goal of this article is to summarize existing research on workplace interventions to prevent disability, relate these to employer disability management practices, and recommend future research priorities. *Methods* The authors participated in a year-long collaboration that ultimately led to an invited 3-day conference, *Improving Research of Employer Practices to Prevent Disability*, held October 14–16, 2015, in Hopkinton, Massachusetts, USA. The collaboration included a topical review of the literature, group conference calls to identify key areas and challenges, drafting of initial documents, review of industry publications, and a conference presentation that included feedback from peer researchers and a question/answer session with an expert panel with direct employer experience. *Results* Evidence from randomized trials and other research designs has shown general support for job modification, RTW coordination, and organizational support, but evidence is still lacking for interventions at a more granular level. Grey literature reports focused mainly on job re-design and work organization. Panel feedback focused on organizational readiness and the beliefs and values of senior managers as critical factors in facilitating changes to disability management practices. While the scientific literature is focused on facilitating improved coping and reducing discomforts for individual workers, the employer-directed grey literature is focused on making group-level changes to policies and procedures. *Conclusions* Future research might better target employer practices by tying interventions to positive workplace influences and determinants, by developing more participatory interventions and research designs, and by designing interventions that address factors of organizational change.

**Electronic supplementary material:**

The online version of this article (doi:10.1007/s10926-016-9664-z) contains supplementary material, which is available to authorized users.

## Introduction

There is a famous quotation from Stephen Hawking, the accomplished physicist afflicted with amyotrophic lateral sclerosis (ALS): “Disability need not be an obstacle to success.” [1]. Yet globally, work-related disability remains a significant burden on workers, employers and society [1]. Work disability occurs when a person is unable to stay at work (SAW) or return to work (RTW) because of an injury or disease [2]. Workplace interventions to prevent and manage work disability focus on changes in the workplace, including equipment, work design and organization (such as working relationships), working conditions or work environment, and can include occupational (case) management with active stakeholder involvement of (at least) the worker and the employer [3–5]. Interventions designed to target particular evidence-based RTW determinants such as job demands, the attitudes and beliefs of the stakeholders, employer practices, or medical symptoms are expected to have an impact on an employee’s ability to SAW or RTW.

With a goal toward improving future research of employer disability prevention strategies, the authors participated in an invited 3-day conference, *Improving Research of Employer Practices to Prevent Disability*, held October 14–16, 2015, in Hopkinton, Massachusetts, USA. Methods and general proceedings of the conference are described in the introductory article to this special issue [6]. The authors of the present article represented a sub-group tasked with understanding the state-of-the-art in workplace interventions and how these relate to employer practices for managing and preventing disability. We were asked to review the applicable scientific literature, assess its relevance for employer decision-making, compare interventions described in the scientific and employer-directed grey literature, contrast key conceptual and theoretical frameworks, and recommend future research priorities.

The body of scientific evidence for understanding which workplace interventions are effective in work disability prevention (WDP) and management have been summarized previously in multiple Cochrane [4, 5, 7] and non-Cochrane reviews [3, 8, 9]. Cochrane reviews remain the gold standard in medicine, but these may be too restrictive for understanding effective workplace interventions, where randomized and carefully controlled trials are not always feasible. As an alternative, some research groups have proposed evidence-based guidelines for key components of workplace-based intervention and disability management programs using non-Cochrane reviews (e.g., the Seven ‘Principles’ for Successful Return to Work from the Institute for Work and Health [10]), but these recommendations have varying levels of evidentiary support. In the context of supporting the translation of knowledge to practice, we consider whether findings from the scientific literature are reflected in best practice summaries available to employers and other stakeholders and whether scientific studies are addressing questions that might influence their decision-making and practices.

The grey literature (reports, articles and research that is produced by organizations or practitioners outside of the academic publication stream) is not often incorporated into the body of academic evidence but nevertheless may reflect the perceptions of employers and other stakeholders about effective ways to solve disability problems. Comparing work disability management approaches in the grey literature and employed by stakeholders with those of the scientific literature may offer some future directions for research and knowledge transfer. Potential disconnects might be expected because employers may be focused on operational efficiency and organizational practicality, while researchers may be more focused on worker well-being. To identify intervention gaps and new research directions and support the implementation of evidence-based RTW programs, it is important to understand the similarities and differences between the academic and practitioner perspectives.

Therefore, the key questions this paper seeks to answer are: (1) What are the predominant workplace intervention components in the scientific and grey literature and what evidence-based RTW determinants do they address?; (2) How do interventions in the scientific literature compare to recommendations in the grey literature and feedback from an employer stakeholder panel?; and (3) What are the intervention gaps and research opportunities?

## Approach

To answer questions about workplace interventions to prevent work disability we considered four sources of information: a) the results of a systematic review of randomized controlled trials from a previously conducted Cochrane review, b) our own summary of the applicable non-Cochrane systematic reviews, c) a sample of documents from the grey literature, and d) feedback from a special stakeholder panel convened for the Hopkinton conference. This strategy enabled us to assess a broad and representative range of resources and information regarding intervention research and practice.

### Defining a “Workplace Intervention”

We defined workplace interventions for work disability prevention as those focusing on changes in the workplace or equipment, work design and organization (including working relationships), working conditions or work environment, and occupational case management with active stakeholder involvement of (at least) the worker and the employer [3, 11]. Active involvement was defined as face-to-face conversations about return to work between (at least) the worker and the supervisor. In this review, a workplace intervention must contain work changes and stakeholder involvement specifically the employer/supervisor. This definition is a synthesis of the International Ergonomics Association (IEA) definition of ergonomic interventions [12] and the Waddell et al. [13] definition of occupational interventions. Changes in the workplace and equipment include changes in the furniture or the materials needed to perform the work. Changes in the work design and organization include changes in schedules or tasks, training in task performance, and altered working relationships with supervisors and co-workers. Changes in working conditions refer to the financial and contractual arrangements; and changes in the work environment concerning noise, lighting, vibration, etc. As long as the workplace intervention was a structural part of the intervention (with the intention to offer the workplace intervention to all participants in the intervention group), studies with interventions that included more components than described in the definition of a workplace intervention were not excluded. Our definition allowed us to include only interventions that were linked closely to the workplace and that focused on work adaptations or the involvement of stakeholders from the work environment. We did not include interventions that were intended to simulate the demands of work in a laboratory setting, without changes to or involvement of the workplace itself in the RTW process in this review [4, 5].

### Cochrane Review

To identify interventions that have received the highest level of scientific scrutiny, we reviewed 14 randomized controlled trials (RCTs) of workplace interventions [14–38] identified through a previously published Cochrane review [5]. The search strategy in the Cochrane review, which includes search terms, inclusion and exclusion criteria, and a PRISMA diagram of the search process can be found in the original publication [5]. For consistency and ease of comparison, workplace interventions for all four literature sources were defined according to the definition in the Cochrane review [4, 5], which is provided above. Papers were excluded from the Cochrane review if they were focused on primary prevention only (preventing incident cases of sickness absence), a RTW outcome measure was not included, if the intervention involved ergonomics education or posture modification only.

### Non-Cochrane Systematic Reviews

To capture studies conducted using other research designs (e.g., non-randomized controlled trials, before and after designs, cohort studies, qualitative inquiry), and targeting organisation, work group, or department level interventions rather than individual level interventions, we identified and assessed a series of systematic reviews. Since our purpose was to capture a representative sample of the available literature, rather than a systematic assessment of all reviews and studies, the reviews were identified by the scientific team based on their knowledge of the existing literature. An initial set of 12 reviews published between 2010 and 2015 was identified by the group as potentially suitable for our purposes. After an initial evaluation, seven reviews [9, 39–45] were retained and one new review was added (identified through the references of an included paper). Reviews were excluded if there was substantial overlap with the studies from the Cochrane review, or if they did not fit with our definition of workplace intervention (above).

### Grey Literature

Papers or reports in the grey literature were identified by searching the World Wide Web using Google as the search engine. Because there is no existing citation search engine for such grey literature publications, these documents were located from a keyword search using various combinations of the terms “employer”, “disability”, “management”, “policy” and “guidelines”. Thirty-three documents were selected as a representative sample of the grey literature available to, and generally produced by, non-academic stakeholders. The opening article in this special issue [6] provides a detailed description of the search strategy and includes an appendix listing the 33 documents provided by the Hopkinton Conference organizers as a representative sample of freely available grey literature publications. We determined that 16 of the 33 documents [10, 46–61] were most appropriate for our discussion. Most of the included papers did not discuss interventions in the scientific sense, but they did describe recommendations for practice, which we have interpreted as recommended interventions. We excluded papers aimed at primary prevention of diseases or those that did not focus on RTW/work disability prevention as the main goal.

### Special Panel

As part of the Hopkinton conference a special panel was convened to inform the knowledge generation process (see introductory paper for a more detailed description of the conference and panel). Our initial evaluation of the available literature was presented to the panel during the conference. The panel subsequently provided feedback on the approach taken as well as the preliminary findings. The perspective of panel members is incorporated into our discussion and recommendations.

### Analytic Approach

To facilitate this comparison and to provide a framework for our discussion, key information was extracted and summarized in separate tables for each of the three document sources (Cochrane reviews, non-Cochrane reviews and grey literature). The key information to be extracted was determined through group discussion and included intervention/recommendation descriptions, intervention components, and the RTW determinants targeted by the interventions. Members of the team were then paired and assigned a subset of documents from each source to review. For all three document sources we first filled out the tables individually, then paired researchers compared results and telephone meetings were used to address any disagreements about the content, and consensus was achieved through discussion.

Our next step was to compare the summarized information from the individual studies, systematic reviews and grey literature to identify patterns. We first did this by comparing the type and frequency of intervention components reported in each literature source. The workplace intervention components were sorted into five commonly used categories [3, 5, 11–13]: (a) changes to workplace or equipment; (b) changes to work design and organization including working relationships; (c) changes in working conditions (financial/contractual arrangements); (d) changes to the work environment (noise/vibration/etc.); and (e) case management with worker and employer (e.g., face-to-face worker-supervisor communication about RTW).

Next we compared the (recommended) workplace interventions from the three literature sources to assess which were intended to address determinants known to play an important role in RTW/WDP. The evidence-based personal and workplace RTW-determinants we considered were derived from recent book chapters focusing on workplace and individual factors in work disability prevention [62, 63]. Workplace factors included physical job demands, psychosocial job demands, work organization and support (e.g., supervisor/co-worker support) employer’s attitudes, practises and beliefs regarding RTW [62]. Personal factors included worker’s attitudes and beliefs about work disability (expectations, self-efficacy), worker’s behavior regarding RTW (fear avoidance, coping), perceived support by the worker, medical symptoms (e.g., pain, stress, anxiety, and depression) [63].

Our preliminary findings were summarized and presented to a special panel of knowledge experts with direct employer experience who were assembled as part of the Hopkinton Conference (see introductory article in this special issue for a description of the special panel make-up and its methodology [6]). Stakeholder panel commentary was then integrated with the findings from our representative literature samples.

## State of Evidence and Practice

In this section of the paper we will address our first question: What are the predominant workplace intervention components in the scientific and grey literature and what evidence-based RTW determinants do they address? We will also provide a summary of the comments provided by the special employer panel regarding the challenges they saw for the design and implementation of workplace-based interventions.

### Results of the Cochrane Review

The Cochrane review we utilized as our first source of information was conducted to determine the effectiveness of workplace interventions for reducing sick absence among workers on disability leave. The authors found high quality evidence that workplace interventions reduced time to first RTW and the cumulative duration of sick absence. The evidence was strongest for workers with MSK disorders. It was not clear that the interventions had any impact on sick leave recurrence or RTW sustainability. There was no evidence that they were effective for mental health conditions or cancer [5]. “Appendix A as Electronic supplementary material” contains a brief summary of the studies included in the review.

#### Intervention Components Included in the Cochrane Review Studies

Table 1 provides a summary of key components included in the Cochrane RCTs. The most frequent RTW/WDP workplace intervention components included in the studies were changes to workplace and job design as well as work organization, including working relationships (included in 13 of the 14 intervention studies). Most interventions also included some form of case management with the worker and employer (with worker-supervisor communication as a key element) (12 studies) or changes to the workplace or equipment (11 studies). Fewer interventions (9 studies) included changes to working conditions, such as a change to the employment contract. Only six interventions included a component that changed the physical work environment.

**Table 1:** Frequencies of applied workplace intervention components included in the Cochrane review studies

Workplace intervention components	Proportion of studies
Changes workplace or equipment (1–6, 8, 9, 12–14)	11/14
Changes work design and organisation including working relationships (1–5, 7–14)	13/14
Changes in working environment (noise/vibration/etc.) (3, 5, 8, 9, 12, 14)	6/14
Changes to the work conditions (financial/contractual arrangements) (1, 4, 5, 6, 7, 8, 9, 12, 14)	9/14
Case management with worker and employer (face-to-face worker-supervisor communication about RTW) (1, 2, 4–12, 14)	12/14

Numbered studies are described in more detail in Table 2.

#### RTW Determinants Addressed by the Cochrane Review Studies

Many of the known evidence-based personal and workplace RTW/WDP determinants are addressed in the 14 papers included in the Cochrane review. The personal RTW/WDP determinants most frequently addressed include medical symptoms (addressed in 13 of 14 intervention studies), worker behavior regarding RTW (12 studies), and worker attitudes and beliefs about work disability (10 studies). The workplace RTW/WDP determinants most frequently addressed were physical job demands (10 studies), psychosocial job demands (10 studies) and work organization and support (10 studies). In comparison, the RTW/WDP determinants that receive relatively little attention are employer’s attitudes and beliefs (4 studies) and perceived support by the worker (7 studies). Overall, there are more RCTs that evaluate interventions addressing three or more personal RTW determinants (11 studies) than RCTs that evaluate interventions addressing three or more workplace determinants (8 studies). Determinants addressed in the scientific papers included in the Cochrane review are shown in Table 2.

**Table 2:** RTW determinants addressed in the Cochrane review studies

Study	Workplace RTW determinants				Personal RTW determinants			
	Physical job demands	Psycho-social job demands	Work organization and support	Employer attitudes, practises and beliefs regarding RTW	Worker’s attitudes and beliefs about work disability	Worker’s behavior regarding RTW	Perceived support by the worker	Medical symptoms
	Y/N	Y/N	Y/N	Y/N	Y/N	Y/N	Y/N	Y/N
Study 1: Anema [14, 15]	1: Y 2: N	1: N 2: N	1: N 2: N	1: N 2: N	1: Y 2: Y	1: N 2: Y	1: N 2: N	1: N 2: Y
Study 2: van Oostrom [16–18]	Y	Y	Y	Y	Y	Y	Y	Y
Study 3: Arnetz [6, 19]	Y	Y	Y	NA	NA	Y	Y	Y
Study 4: Blonk [7]	NA	NA	NA	NA	Y	Y	Y	Y
Study 5: Bültmann [8]	Y	Y	Y	Y	Y	Y	Y	Y
Study 6: Lambeek [9–11]	Y	Y	Y	Y	Y	Y	Y	Y
Study 7: Busch and Jensen [12, 24]	N	N	N	N	Y	Y	N	Y
Study 8: Hees [26, 27]	Y	Y	Y	Y	Y	Y	y	Y
Study 9: Vlasveld [28, 29]	N	Y	N	N	Y	Y	Y	Y
Study 10: Loisel [30–32]	Y	N	Y	N	N	N	N	Y
Study 11: Noordik [33, 34]	N	Y	Y	N	Y	Y	N	Y
Study 12: Tamminga [35, 36]	Y	Y	Y	N	Y	Y	N	Y
Study 13: Verbeek (2002)	Y	Y	Y	N	N	Y	N	Y
Study 14: Feuerstein [38]	Y	Y		N	N	N	N	N
Total proportion	10/14	10/14	10/14	4/14	10/14	12/14	7/14	13/14

### Results from Non-Cochrane Systematic Reviews

Research conducted at the work group, department or organizational level that was included in the non-Cochrane systematic reviews tended to address workplace determinants more commonly than personal determinants. The most frequently included intervention component identified in the studies assessed by these reviews is case management with worker and employer communication regarding RTW (found in 6 of the 8 reviews). Changes to workplace equipment (5 reviews), work design or organization (5 reviews) and working conditions (4 reviews) were also common. Interventions with components that change work relationships (3 reviews) or financial and contractual arrangements were least common (2 reviews). Table 3 summarizes intervention components and RTW determinants addressed by the studies in the reviews. The most frequently addressed RTW determinants were work organization and support (found in 6 of the 8 reviews) and physical job demands (5 reviews). The least frequently addressed RTW determinants are psychosocial job demands (2 reviews), worker attitudes and beliefs (2 studies), worker behavior (2 reviews) and perceived support (2 reviews).

**Table 3:** RTW determinants and intervention components in non-Cochrane systematic reviews

	Intervention components included changes to:	Workplace RTW determinants				Personal RTW determinants
Study	(a) workplace or equipment	Physical job demands	Psychosocial job demands	Work organization and support (supervisor/coworker support)	Employer’s attitudes/practices and beliefs regarding RTW	(a) worker’s attitudes and beliefs about work disability (expectations, self efficacy)
	(b) work design or organization					(b) worker’s behavior regarding RTW (fear avoidance, coping)
	(c) organization including working relationships					(c) perceived support by the worker
	(d) changes to work environment (noise/vibration/etc.)					(d) Medical symptoms (e.g., pain, stress, anxiety, depression)
	(e) work conditions (financial/contractual)					
	(f) case management with worker and employer (face-to-face worker-supervisor communication about RTW)					
Carroll [39]	a, b, c, d, e, f,	Y	Y	Y	N	c, d
Furlan [40]	f	N	N	Y	N	none
Gensby [9]	a, b, c, d, f	Y	N	Y	Y	none
Odeen [41]	a, b, d	Y	Y	Y	N	a, b, d
Palmer [42]	a, b, c, d	Y	Y	Y	Y	none
Pomaki [43]	f	N	N	N	N	c, d
Schandelmaier [44]	f	N	N	Y	N	c
Nevala [45]	a, b, c, d, e, f	Y	N	Y	Y	a, b

Six systematic reviews focused on interventions that targeted primarily musculoskeletal disorders (MSDs). Two reviews specifically sought workplace intervention research targeting mental health conditions. The MSD studies will be discussed first. Carroll et al. [39] conducted a systematic review to determine if interventions involving the workplace are more effective for RTW than interventions that do not have a workplace component. They identified 9 studies that primarily assessed interventions for low back pain, and concluded that simply involving the workplace is not enough. Palmer et al. [42] and Schandelmaier et al. [44] drew similar conclusions, suggesting that RTW coordination (collaborative planning among stakeholders to implement work modifications) is more consistently effective than other intervention types. Similarly, Gensby et al. [9] assessed the nature and effectiveness of workplace disability management programs on RTW. They found 13 studies, but concluded there was insufficient data to determine if the programs overall (or specific components of them) are effective. Gensby et al. also found that most interventions targeted MSK injuries and were conducted in blue collar or health care sectors. Nevala et al. [45], focused their review more narrowly on workplace accommodation as the intervention, but included a broad range of health conditions and study designs. They concluded that there is some evidence that specific types of work accommodations (such as vocational counselling, changes to work schedules and work organization) are effective for workers with physical disabilities, but there is less evidence supporting work accommodation for cognitive disabilities. Finally, Odeen et al. [41] assessed the effectiveness of “active” workplace-based interventions on RTW. Active interventions are those that encourage activity and where the goal is behavioral change. Seventeen studies were included in a qualitative synthesis. The authors found limited evidence that active interventions are not generally effective in RTW. However, there was some evidence that graded activity, the Sherbrooke model and CBT could reduce work absence. They concluded that only interventions involving consultation and consensus between stakeholders combined with subsequent work modification offers consistent, positive results.

Furlan et al. [40] and Pomaki et al. [43] assessed workplace-based interventions targeting mental health conditions. Furlan et al. [40] looked specifically at interventions for depression. Twelve studies were identified, but the quality of evidence was low. This was primarily because the studies had a high risk of bias and there were few studies that assessed similar outcomes, which affected consistency and precision of evidence. The authors concluded that no single intervention targeting depression could be recommended as effective in RTW. Pomaki et al. [43] considered a broader range of mental health conditions. They found 8 studies and concluded that facilitating access to clinical treatment and workplace-based high intensity psychological interventions improve work function, quality of life, and reduce costs associated with common mental health conditions. The evidence suggesting these interventions reduce absence was limited.

### Grey Literature

The documents from the grey literature were of three broad categories: a) reports on case studies of employer organizations and business networks on managing general disability/chronic illness, b) reports on the cost/benefit of RTW programmes, corporate policies/programs for disability management and RTW, or c) international, national or regional codes/guidelines for RTW policies and RTW-guides from insurers. The audience targeted by the grey literature was generally human resource managers, disability, health and productivity managers, physicians, and/or RTW-coordinators and their teams.

Table 4 summarises the intervention components that were recommended in the grey literature. We found that in the included reports, RTW/WDP recommendations focused mainly on work/job design and work organisation (9 of the 16 reports). The predominant recommendations are the facilitation of the employee’s gradual return to work and the identification/provision of modified and transitional duties (10 reports). There is limited focus on workplace and equipment design with only three reports recommending changes to workplace/equipment design, with two of these reports specifically recommending ergonomic assessments. There is some (although limited) focus on RTW/WDP recommendations for case management with worker-supervisor communication (4 reports). The reports mainly highlight early and continuing contact with the worker during sick leave and RTW (3 of the 4 reports). RTW/WDP recommendations regarding working conditions (noise/vibration/etc.) and work environment (financial/contractual arrangements) are not mentioned at all in the included papers from the grey/employer literature

**Table 4:** Frequencies of recommended workplace interventions in the grey/employer literature, classified by Cochrane review categories

Recommended workplace intervention components	Proportion of reports (n = 16)
Changes workplace or equipment design	3/16
Provide adaptations with input from the worker and with technical expertise [17]	
Provide ergonomic assessments [27]	
Incorporate ergonomic assessments [29]	
Changes work/job design and organisation including working relationships	9/16
Have a policy to make a routine offer of modified duty [13]	
Support worker while not disadvantaging co-workers and supervisors [13]	
Provide modified work options [15]	
Identify transitional work opportunities [18]	
Develop a list of transitional duties [19]	
Make more effective use of job descriptions in the RTW process [20]	
Acknowledge and deal with normal human reactions [22]	
Update and analyze job descriptions [25]	
Create transitional RTW and prevention programs [25]	
Provide a supportive work environment [27]	
Provide more opportunities for transitional/limited duty positions [27]	
Create a “transitional work fund” [27]	
Implement a structured transitional work program that can provide effective RTW options and accommodation for both work-related and non-related problems [29]	
Changes in working environment (noise/vibration/etc.)	0/16
Changes to the work conditions (financial/contractual arrangements)	0/16
Case management with worker and employer (face-to-face worker-supervisor communication about RTW)	4/16
Employer makes early and considerate contact with injured/ill workers [13]	
Employer contact should begin early and continue often through duration of the employee’s disability absence [18]	
Maintaining supervisor communication with your employee, WCB case worker and health care providers [19]	
Improve communication with employees about RTW [20]	

See extract of recommendations in the grey literature provided by Bill Shaw: numbers are referring to the papers included in the grey literature review

In the grey literature, evidence-based workplace determinants are reported approximately twice as often as evidence-based personal RTW determinants. Table 5 shows the types of evidence-based workplace determinants that were most frequently addressed in the grey literature: work organizational factors (15 of the 16 reports), physical job demands (13 reports), and employer RTW attitudes, practises and beliefs (12 reports). There is relatively less attention in the included grey literature for psychosocial job demands (9 reports). The evidence-based personal factors that are mentioned most frequently in the papers are medical symptoms (8 reports). Less attention is focused on worker’s RTW attitudes and beliefs (6 reports), worker’s perceived support (4 reports) and RTW behavior (4 reports).

**Table 5:** RTW determinants mentioned in the grey literature

Document*	Workplace factors				Personal factors			
	Physical job demands	Psycho-social job demands	Work organization and support	Employer’s attitudes/practises and beliefs regarding RTW	Worker’s attitudes and beliefs about work disability	Worker’s behavior regarding RTW	Perceived support by the worker	Medical symptoms
Article 1 [46]	Y	Y	Y	Y	Y	Y	N	Y
Article 12 [47]	N	N	Y	Y	N	N	Y	N
Article 13 [10]	Y	N	Y	Y	N	N	Y	N
Article 14 [48]	N	N	Y	Y	N	N	N	N
Article 15 [49]	Y	Y	Y	Y	Y	Y	Y	Y
Article 16 [50]	Y	Y	Y	Y	N	N	N	N
Article 17 [51]	Y	Y	Y	Y	N	N	N	N
Article 18 [52]	Y	N	Y	Y	N	N	N	N
Article 19 [60]	Y	N	Y	Y	Y	N	N	N
Article 20 [53]	N	N	N	Y	N	N	N	Y
Article 22 [61]	Y	Y	Y	Y	Y	Y	Y	Y
Article 23 [54]	Y	N	Y	N	N	N	N	Y
Article 24 [55]	Y	Y	Y	N	N	N	N	Y
Article 25 [56]	Y	Y	Y	N	N	N	N	Y
Article 27 [57]	Y	Y	Y	N	Y	NA	NA	Y
Article 29 [58]	Y	Y	Y	Y	Y	Y	N	NA
	13/16	9/16	15/16	12/16	6/16	4/16	4/16	8/16

### Special Panel Contribution

We identified five overarching themes in their discussion of RTW and disability management:

#### *A Gap in Translation Science*

The panel members raised the concern that results from research are not translated for implementation in organisations. This gap prevents organisations from focusing on factors that contribute to work disability, and utilizing the interventions that research has identified as effective. This often occurs because practitioners do not know the status of the research. A number of strategies were suggested for knowledge translation in protocols for research projects. Research also needs to be reformulated for communication to a non-research audience. For example, panellists suggested that stakeholders would prefer presentations consisting of a few slides that include the cost-effectiveness of the intervention, rather than lengthy reports. Another issue the panel raised was the need to develop researcher competence in knowledge translation and presentation to non-academic audiences. Researchers may need to adopt persuasive tactics rather than the more traditional academic approach. The panellists suggested that the carefully articulated limitations and generalizability in academic presentations, may reduce the impact of messages about intervention effectiveness for practitioner audiences.

#### *The Value of Personal Stories from Management Networks*

Related to the concept of knowledge translation, the panel identified the importance of personal stories in persuading organizational leaders to take action. Personal stories told by trusted individuals in the social networks of organizational leaders are often used to decide how best to deal with disability in the workplace. The panel suggested that researchers’ use the form of case-based stories when they present research to organisations, as this is commonly used in management education and well-known to business leaders. It also requires that researchers become trusted partners in the networks of business leaders.

#### *Beliefs and Values of Organizational Leaders*

Corporate decisions are often based on the beliefs of senior leaders, which become entrenched in workplace cultures. The focus is on meeting financial objectives, and the need to respond quickly to environmental threats. This means that the normative positions of leaders guide decision-making about disability management, and decisions may be emotional, reactive or reflect other business priorities rather than research evidence. Organisations want to perform according to best practice, but implementing work disability interventions must be important to leaders and cost effective. Moreover, it may be that to move past resistant belief systems about disability prevention and management, an “organisational crisis” (e.g., a serious accident or legislative change) is necessary. Researchers and organizations need to figure out how to implement effective interventions without the need for crises, and in the face of potential resistance, financial constraints and environmental complexity.

#### *Organizational Readiness*

As a parallel to the individual measures of readiness for change in lifestyle (e.g., smoking cessation, weight management), the panel suggested researchers should start by measuring organizational readiness to change before trying to implement interventions in organisations. While there is a body of research in managing organizational change (which includes measures of readiness), there is a gap between this body of knowledge and disability management research, which is dominated by scholars in medicine. Collaborative efforts between organizational and disability management scholars are needed to close this gap.

#### *Efficacy Versus Effectiveness*

The panel also pointed to the problem that interventions designed and implemented in organizations for a research study, where they can be carefully implemented, monitored and followed-up by the researchers, may be more likely to show results than when those same interventions are implemented without such support. Disability intervention researchers need to work with organizations to implement and support sustained use of effective interventions.

In addition to the five themes, the panel also pointed to the importance of de-medicalization, a shift in focus from the medical aspects of disease or illness to the functional abilities of the employee. It is important to understand both what an employee can and cannot do. Once the focus is on function rather than on the medical aspects, the workplace and supervisors are more natural collaborators in the development of the individual RTW process. The considerations and decisions are no longer just up to the physician, the workplace is recognised as important and performance management can replace disability management. One suggestion of the panel was to do outreach work with physicians to have them visit the workplace to understand the work and thus be able to suggest suitable accommodations that are beneficial to the employee, and sustainable at the workplace.

Finally, the panel considered the employees’ own engagement in the RTW process as another important issue. Employees with an active engagement in their RTW process are considered more likely to have a successful RTW process.

### Differences and Similarities

Our second objective was to compare workplace interventions studied in the scientific literature with interventions recommended in the grey literature and comments provided by the panel, to understand similarities and important differences that may be useful in guiding future research. In conducting this analysis, we also found distinctions between the Cochrane studies and the non-Cochrane reviews. We note them here, though these are not the focus of our analysis. For example, the Cochrane studies were most often conducted at the individual level and included only RCTs. The non-Cochrane reviews included studies at the organization or group level and reflected a broader range of research methods (e.g., non-randomized controlled trials, cohort studies, qualitative inquiry).

#### *Intervention Component Similarities*

Both the grey and scientific literatures recommend or test intervention components that include changes to job design or the organization of work. Recommendations in the grey literature focus mainly on identification/provision of modified and transitional duties. These intervention components have also been frequently tested in the scientific literature (13/14 Cochrane studies and 5/9 non-Cochrane reviews). The modification of job duties to support RTW and prevent work disability therefore appears to be well-investigated by the scientific community and recognized as best practice in the grey literature.

Another interesting similarity between the scientific and practice literature is that intervention components rarely include changes to the physical work environment (noise/vibration, etc.) or working conditions (financial/contractual arrangements). This may reflect a gap in both research and practice for WDP/RTW, or it may be that interventions addressing these issues were not captured in the literature reviewed. Interventions that change the physical work environment may be discussed or assessed in engineering or occupational health and safety literature rather than the medical and disability management streams. Similarly financial/contractual intervention components may be assessed elsewhere, or they may be less common because employment terms and conditions are regulated by law and not easily manipulated in research or in practice.

#### *Intervention Component Differences*

The scientific literature quite frequently assesses interventions that alter workplace equipment. Eleven Cochrane studies and five non-Cochrane reviews include papers assessing this intervention component. Only grey literature papers recommend this and it was not discussed by the panel. When it is addressed in the grey literature, the main recommendation is to provide ergonomic assessment from a qualified expert. This difference is perhaps not surprising since the grey literature mostly explores process recommendations involving experts that result in changes. In this case an ergonomic assessment is likely to result in alterations in workplace equipment.

A difference that is perhaps more notable because it falls within the workplace practice domain relates to case management. One of the most predominant intervention components we found in the Cochrane studies was case management with worker-supervisor communication as a key element in case management. These interventions are most often found in MSK-related research rather than in mental health or cancer research. Case management is not only used independent of diagnosis, but also across different social security systems and settings. In comparison, employer/health care provider communication with worker (early contact and continuity of contact) are mentioned in only four of the grey literature papers. As noted above, the grey literature focused almost entirely on changes to job design and work organization.

#### *RTW/WDP Determinant Similarities*

In comparing the scientific literature to the grey literature and panel feedback on these criteria, the greatest similarity is the focus on interventions that target physical job demands and work organization and support. More than one third of the Cochrane review interventions addressed physical job demands and work organization. Both were addressed in 10/14 studies. In the non-Cochrane systematic reviews physical job demands were captured by the studies in 5/8 systematic reviews, and work organization and support were captured by the studies in 7/8 systematic reviews. The interventions in the grey literature also addressed these determinants quite frequently (physical job demands = 13/16, work organization and support 15/16). Similarly, the panel acknowledged that accommodating workers through changes to physical job demands and changes to the organization of work were common and generally required by law in most countries.

The scientific and grey literature were also similar in that little attention was paid to perceived support from supervisors and co-workers for returning workers. Only 4/16 grey literature papers, 7/14 Cochrane review studies and 3/8 non-Cochrane systematic reviews addressed this determinant. This is an important determinant and our analysis suggests that it requires greater attention in both research and practice.

#### *RTW/WDP Determinant Differences*

We found that in the Cochrane studies the personal determinants of worker behavior, medical symptoms, and RTW beliefs/expectations were addressed most frequently by the interventions being tested. The non-Cochrane reviews addressed workplace determinants more often than personal RTW determinants. Three of the reviews included studies that did not address any personal determinants. In comparison, the grey literature mentions workplace factors twice as often as personal factors. Moreover, the grey literature attends to employer attitudes/practices and beliefs about RTW in 12/16 papers. Research assessing employer attitudes as a determinant was noticeably lacking in both the Cochrane and the non-Cochrane reviews. This is particularly concerning when a key theme in the feedback from the panel is the importance of organizational attitudes, cultures and leader beliefs.

A final point of comparison we would add is that in most of the grey literature included in this review, the main message is about productivity: reducing disability costs and increasing profit (e.g., articles 2, 12, 21). From an organizational point of view disability in a worker may threaten the worker’s performance, which is one important element of organizational success. This perspective is reflected in papers that provide cost/benefit analysis to make the “business case” for disability management programs (e.g., articles 7,16, 23). This productivity perspective is considered in many of the non-Cochrane reviews, which are more likely to measure productivity and performance outcomes. However, it is in stark contrast to the perspective in the Cochrane studies, which is about the disabled worker and his/her welfare. RTW interventions from the scientific literature are often developed by medical, disability and health psychology researchers and the research is focused on improving health and quality of life for the individual. Interventions are designed to help the worker return to work because this is what the prevailing paradigm says is good for the worker, not because it is good for the economy, society, or the company. These different perspectives are challenging, but provide insight into the need for new directions in research and knowledge translation.

### Intervention Gaps

#### Intervention Components

Workplace intervention components in the scientific (Cochrane and non-Cochrane reviews) and grey literature mainly concern changes to workplace design, job design, and work organization. They tend to address factors that are easier to modify in the workplace, such as physical job demands (in comparison to psychosocial or organizational components). Following the panel recommendations, there needs to be greater emphasis on other components at the group, organizational and leader levels. For example, the Cochrane papers indicate that supervisor and coworker involvement in the RTW process may be helpful, but they are not often included in the interventions considered by employers [64]. The supervisor may play a pivotal role in facilitating job change and supervisor/coworker support may be important RTW facilitators. Since supervisor/coworker engagement in RTW best practice recommendations is rare, strategies have to be found to enhance their engagement.

The finding that identification/provision of modified and transitional duties (job design and organization) is the predominant RTW recommendation in the grey literature, aligns with employer interest in work organisation and physical job demands. However, it may be that the origin of this recommendation is more legally determined than evidence-based. This follows the logic of the panel, who suggested that organizational decision-making may be influenced by “crisis” or response to local regulatory factors. In many jurisdictions there are legal requirements for employers managing workers with temporary or permanent work disability. For example, in Canada, human rights law requires that employers accommodate workers with disabilities up to the point where it causes the employer undue hardship [65]. The legal requirement in many countries is similar. In the U.S., the employer ‘must be open to returning an injured employee to work if he/she (a) can continue to perform the essential functions of the assigned job with reasonable accommodation, or (b) are qualified to perform another available job with or without reasonable accommodation.’ [66]. Although the specific approach and application (public vs. private sector), differs across countries, most anti-discrimination law requires that employers make accommodations and alter job profiles in order to support the employment of workers with disabilities. An important implication of this is that researchers need to examine the types of accommodations that are most effective (for both employer and employee), for differing medical conditions and job types.

Another reason grey literature recommendations target job design and work organization (modified duties, light duties) may be pragmatic. Provision of modified or light duties is transitional by nature, costs are usually low and they are directly applicable/available in many companies [4, 67–69]. Furthermore, the employer has expertise in work design for their particular operations. They concentrate their efforts on factors under their control and within their domain of experience. This means less attention is paid to psychosocial and cultural issues which are less controllable, and for which organizational benefits are more difficult to measure. This may also explain why RTW recommendations for ergonomic changes to workplace or equipment design are very limited in the grey literature. Practically speaking, the purchase of ergonomic equipment for a sick listed worker may be costly, particularly for a small organization. However, if it must be purchased as a legally required accommodation, the ergonomic “prescription” is often made by specialists from outside of the organization. Employers may perceive that ergonomics, like medication or treatment prescription, is beyond their expertise. This highlights the need for multidisciplinary, stakeholder involved approaches to intervention design. Each party brings expertise that can be combined to develop innovative solutions.

Finally, a subtle distinction is that the scientific literature recommends early and continued face-to-face supervisor-worker communication, this intervention component is rarely noted in the grey literature reviewed here, though the panel recommendation for worker inclusion suggests there is awareness that this is important. Instead of direct supervisor-worker communication, the grey literature recommends assigning a RTW coordinator to guide case management with both internal and external stakeholders/providers (e.g., 19, 27). The preference for a RTW coordinator may occur in a workers compensation claim where a RTW plan is required and must be coordinated among all involved parties i.e. the injured employee, the injured employee’s supervisor and health care provider, and union representatives (if workplace is unionized) [70].

#### RTW Determinants

Overall, there appears to be a larger gap between the Cochrane review studies and the grey literature than there is between the non-Cochrane research and the grey literature. It is perhaps not surprising that interventions addressing personal (bio-psycho) determinants rather than workplace (social) determinants are found more frequently in the RCTs that were included in the Cochrane review studies [5]. This may have occurred for several reasons.

First, it may be that the interventions target determinants that are easier to measure and modify. It is easier to ensure adequate controls are in place, and to compare interventions when there are fewer intervention components and where the outcome measure and intervention can be more directly linked to personal factors rather than contextual factors. For example, the study by Feuerstein et al. [38] includes two components: (1) an ergonomic assessment, and (2) training to improve problem-solving. The study measures the impact on the worker’s satisfaction with treatment, pain, symptoms and function, general health, problem-solving and work absence. By comparison, studies targeting social determinants and conducted at the organizational or group level are more challenging to design. Workplaces are large, open systems influenced by the competitive, economic and legal contexts in which they operate. The social environment within the organization may change rapidly as a result of factors (e.g., a change in leadership, merger or acquisition) that have little to do with any planned interventions. This makes the social context particularly difficult to measure and interventions difficult to design. For example, Gensby et al. [9], in their systematic review assessing the nature and effectiveness of workplace disability management programs on RTW were able to find 12 studies examining 10 disability management programs. The authors noted that the high risk of bias in the studies made it difficult to draw strong conclusions regarding the effectiveness of the programs overall (or specific components of them). Information on sample characteristics and effect sizes was uncertain and environmental complexity affects the ability to determine causality. To conduct research that would be considered as rigorous as the RCTs in the Cochrane review [5], researchers would need to develop and secure funding for complex, longitudinal observational, and field studies that theoretically justify how a single intervention (or group of interventions) would have a sustained impact on the organization or the social behavior of its employees.

Second, the focus on personal factors in the Cochrane review [5], such as symptoms and expectations (bio-psycho), may reflect the fact that most of the studies were conducted by researchers from medical disciplines. Since the primary expertise of medical researchers is illness or injury rather than the workplace, the research is aimed at the ill or injured worker. This results in more research regarding the reduction of medical symptoms for individuals and their particular health conditions, addressing physical demands to prevent exacerbation or re-injury for the individual, and the worker’s individual psychological response to their illness, injury, treatment and RTW.

The focus on workplace factors in the grey literature might also have several explanations. First, workplace factors related to sick leave or workplace barriers for RTW are easier to identify for the employer and easier to modify than personal worker factors. Second, many employers hesitate to discuss personal factors like the worker’s RTW attitude and behavior because the conversations may be uncomfortable and employers may feel it is an invasion of privacy. Third, employers also have limited knowledge of, or access to, the employee’s medical information so it is difficult to tell whether the behavior is related to the illness/injury and RTW or whether it is occurring for some other reason. Conversations regarding a returning employee’s difficult behavior may become disciplinary in nature, which then raises a number of legal concerns for employers and insurers [71].

It is interesting that when personal work disability determinants were considered in the grey literature, medical factors received the most attention. This is in contrast with the panel’s suggestion that there needs to be a “de-medicalization” of RTW research, but is aligned with a stakeholder belief that duration of sick leave and RTW is mainly determined by the duration of medical/physical symptoms [71]. This suggests that RTW/SAW interventions are primarily driven by the dominant medical work disability paradigm rather than the psychosocial paradigm. Employers pay attention to worker’s physical symptoms and physical job demands as barriers to RTW. In contrast, psychosocial factors receive less attention. Also the relationship between psychosocial issues and work disability is less clear. Despite the call for de-medicalization, organizational leaders (and other stakeholders) may still believe that disability management and prevention belongs to the medical community and is their problem to solve by finding the cure for whatever ails the workforce.

Overall, it is clear there are gaps between medical research (Cochrane review) and the grey literature with respect to the disability determinants they address. However, this seems to be partly bridged by the organizational and unit level interventions we found in the non-Cochrane reviews. In particular, the non-Cochrane reviews show that scientists are designing workplace interventions that address the determinants of work organization and support as well as employer attitudes, practices and beliefs. Nonetheless, there is clearly a need for more cross-disciplinary research and knowledge exchange about all RTW determinants of the bio-psychosocial model in order to most effectively prevent and manage work disability.

## Research Opportunities: Towards a Common Understanding and Call to Action

Do we care if our research gets implemented in practice? If the answer is “yes”, then we need to understand what the employer and other stakeholders are interested in achieving. Based on our simple contrast of scientific literature, grey literature and panel feedback we see two cultures with the most tenuous of connections: a culture of science and a culture of practice. Organizational practices and policies are important for return to work among injured workers [72, 73]. In many cases, organizational management knows which practices and policies to follow, but does not follow them because of underlying contextual issues that shape what gets done. For example, disability will be managed differently in a culture of operational efficiency than a culture of integrated health, safety and operations [74]. If supervisors and managers are only incentivized operationally, we should not be surprised to see limited implementation of RTW policies that require supervisor engagement. Changing organizational culture is difficult and takes a long time [75]. Keeping everyone in the organization committed to a change for the time it takes the new practices to take hold, is a major challenge in this kind of work [75]. If stakeholders, including researchers, want to improve RTW and SAW practice in organizations, we need to rethink our methods, interventions, and processes.

### *Intervention Methods*

The systematic reviews we have relied on in this paper have concluded that much of the research being conducted is of a very high quality, and that evidence is mounting with respect to factors that contribute to work disability and interventions that may increase the likelihood of a sustained RTW. However, the methods being used by scientists do not integrate the organizational context in a way that helps us understand when, and in what circumstances our interventions are most likely to be successful. This is a problem that has been faced by organizational researchers for decades, and we should look to their experiences to guide future research in ways that balance rigor with reality. A recent commentary on the state of research methods in organizational studies, states:

Perhaps the single biggest reason why there are so few true experimental designs used in evaluation of organizational change interventions is that they are, without being too simplistic about it, really, really difficult to execute. While it is not impossible to use random assignment, the opportunities to do so are very limited…The field is unlikely to magically discover how to routinely use true experimental designs in evaluation research given most organizational realities. [76] (reprinted with permission).

To strengthen the tenuous link between research and practice we need to be designing studies that ensure validity but use alternative designs. Research must be collaboratively developed with stakeholders, which means it must be co-designed, co-conducted and co-evaluated [76]. We need to move past the focus on personal RTW determinants and design interventions at the individual, group and organizational level. This would mean using multilevel modelling, longitudinal (time-series) analysis, and quasi-experimental research.

### *Intervention Content*

Perhaps the most audacious call we can make is for medical researchers to stop designing medical interventions for workplaces. The principles and approaches of the burgeoning field of positive psychology offer an interesting way to think about designing workplace interventions that address disability by embracing it as a normal part of aging and the human condition. Positive psychology focuses on the scientific study of strengths and social structures that enable individuals to thrive [77, 78]. In terms of work disability, positive interventions would encourage us to think beyond the prevention of illness or injury and repairing the damage it causes. A positive approach would seek to identify what strengths arise from work disability, what new opportunities are created for employees and their employers, what skills and abilities can be enhanced by experiences with disability. The intention is not to ignore the negative impact of work disability; the body matters, pain and ability limitations matter. But when we focus only on the negative we perpetuate a belief that employees with disabilities are not whole and a burden that must be borne by the employer and society. We have to move past the idea of accommodating workers with disabilities and toward interventions that enhance and develop new strengths in employees whose health status evolves over time.

The design of interventions should also be participatory and address the social context. This means collaborating with workers, employers and other stakeholders to design interventions that are realistic, financially feasible, and appropriate for the work that must be done. Researchers cannot do this without the input of employees and employers. Inclusiveness means helping employers design safe, inclusive jobs from the start. Good examples of inclusive research designs can be found in the participatory ergonomics literature [79–81] and the occupational health field [82, 83]. We should also be investigating the effect of interventions like job rotation, job carving (task restructuring), job crafting (employee-driven change) and skill enhancement on health and productivity. Finally, we need to design interventions that target or at least take into account the worker’s social environment. We cannot ignore the impact of workplace culture, employment history, social networks, power, politics and conflict.

### *Intervention Process*

One of the most important discoveries from the Hopkinton conference is that organizational stakeholders are interested in implementing interventions, but need more than evidence that they are efficient and effective. They also need researchers to identify how they can successfully implement health and safety interventions and ensure sustainability to improve disability outcomes. We feel that to ensure interventions are effective over the long term we should be incorporating principles of change management in our research designs. Organizational change management has been a field of study since the 1970 s, and addresses how change can be managed effectively [84]. It entails interventions that are intended to influence “the task-related behavior and associated results of an individual, team or entire organization” [84]. While not without its problems [84], the field has provided a number of models that offer a starting point for researchers seeking to build change management processes into their study designs. A review by Armenikas and Bedeian [85] offers a systematic integration of change management theory that addresses content, context and process [85]. Readiness for change and sustainability are key components in change management and emerging areas of interest for organizational scholars. Readiness for change in particular is being explored from an institutional perspective [86], and at the micro-, meso- and macro-levels within organizations [87]. Implementation issues are addressed in more detail in a companion article within this special issue [88].

We were asked to compare intervention research with practitioner recommendations for improving work disability management and outcomes for employees. We found an impressive body of scholarship that had some, but not enough ties to the workplace. The approach we took has some strengths and limitations. The wide range of literature sources we used and the Hopkinton “think tank” process, including the expert panel, critique and dialogue were a unique approach that contributed to the quality of our analysis. The grey literature we relied on is limited by the use of Google as a search engine which makes replication difficult. Furthermore, the grey papers were primarily produced by employer, insurer, national and international organizations. Thus the grey literature lacked the perspective of the workers themselves who are integral to the effectiveness of any RTW/WDP intervention. Although worker perspective was captured in much of the scientific literature our analysis would have benefited from the expertise of a worker representative on the special panel. “*Nothing about us without us*” is a pithy statement frequently heard in disability circles, but it is perhaps one that ought to be taken to heart by researchers. We cannot do this well on our own; it is our view that inclusiveness in research design and execution is the necessary path forward.

## Electronic supplementary material

Below is the link to the electronic supplementary material.

Supplementary material 1 (DOCX 35 kb)

## References

[1] WHO. World report on disability. World Health Organization; 2011. 26131540

[2] Loisel P, Anema J (2013). Handbook for work disability: prevention & management.

[3] Franche R, Cullen K, Clarke J, Irvin E (2005). Workplace-based return-to-work interventions: a systematic review of the quantitative literature. J Occup Rehabil.

[4] van Oostrom SH, Driessen MT, de Vet HC, Franche RL, Schonstein E, Loisel P (2015). Workplace interventions for preventing work disability (review). Cochrane Database Syst Rev.

[5] van Vilsteren M, van Oostrom SH, de Vet HC, Franche RL, Boot CR, Anema JR (2015). Workplace interventions to prevent work disability in workers on sick leave. Cochrane Database Syst Rev.

[6] Shaw WS, Main CJ, Pransky G, Nicholas MK, Anema JR, Linton SJ (2016). Employer policies and practices to manage and prevent disability: foreward to the special issue. J Occup Rehabil.

[7] Aas RW, Tuntland H, Holte KA, Røe C, Lund T, Marklund S (2011). Workplace interventions for neck pain in workers (review). Cochrane Database Syst Rev.

[8] Williams-Whitt K, White MI, Wagner SL, Schultz IZ, Koehn C, Dionne CE (2015). Job demand and control interventions: a stakeholder-centered best-evidence synthesis of systematic reviews on workplace disability. Int J Occup Environ Med.

[9] Gensby U, Labriola M, Irvin E, Amick Iii BC, Lund T (2014). A classification of components of workplace disability management programs: results from a systematic review. J Occup Rehabil.

[10] Institute for Work & Health. Seven ‘principles’ for successful return to work. http://www.iwh.on.ca/seven-principles-for-rtw. Institute for Work & Health, 2014.

[11] Anema J (2004). Low back pain, workplace intervention and return-to-work.

[12] Stapleton C, Stapleton C (2000). Classification scheme. Ergonomics abstracts.

[13] Waddell G, Burton A (2001). Occupational health guidelines for the management of low back pain at work: evidence review. Occup Med.

[14] Anema J, Steenstra I, Urlings I, Bongers P, De Vroome EMM, Van Mechelen W (2003). Participatory ergonomics as a return-to-work intervention: a future challenge?. Am J Ind Med.

[15] Anema J, Steenstra I, Bongers P, De Vet H, Knol D, Loisel P (2007). Multidisciplinary rehabilitation for subacute low back pain: graded activity or workplace intervention or both? A randomized controlled trial. Spine.

[16] Van Oostrom S, Anema J, Terluin B, De Vet H, Van Tulder M, Van Mechelen W (2008). Cost-effectiveness of a workplace intervention for sick-listed employees with common mental disorders: design of a randomized controlled trial. BMC Public Health.

[17] Van Oostrom S, Heymans M, De Vet H, Van Tulder M, Van Mechelen W, Anema J (2010). Economic evaluation of a workplace intervention for sick-listed employees with distress. Occup Environ Med.

[18] Van Oostrom S, Van Mechelen W, Terluin B, De Vet H, Knol D, Anema J (2010). A workplace intervention for sick-listed employees with distress: results of a randomised controlled trial. Occup Environ Med.

[19] Arnetz B, Sjogren B, Rydehn B, Meisel R (2003). Early workplace intervention for employees with musculoskeletal-related absenteeism: a prospective controlled intervention study. J Occup Environ Med.

[20] Blonk R, Brenninkmeijer V, Lagerveld S, Houtman I (2006). Return to work: a comparison of two cognitive behavioral interventions in cases of work-related psychological complaints among the self-employed. Work Stress.

[21] Bültmann U, Shersen D, Olsen J, Hansen C, Lund T, Kilsgaard J (2009). Coordinated and tailored work rehabilitation: a randomized controlled trial with economic evaluation undertaken with workers on sick leave due to musculoskeletal disorders. J Occup Rehabil.

[22] Lambeek L, Anema J, Van Royen B, Buijs P, Wuisman P, Van Tulder M (2007). Multidisciplinary outpatient care program for patients with chronic low back pain: design of a randomized controlled trial and cost-effectiveness study. BMC Public Health.

[23] Lambeek L, Van Mechelen W, Knol D, Loisel P, Anema J (2010). Randomised controlled trial of integrated care to reduce disability from chronic low back pain in working and private life. Br Med J.

[24] Busch H, Bodin L, Bergstrom G, Jensen I (2011). Patterns of sickness absence a decade after pain-related multidisciplinary rehabilitation. Pain.

[25] Jensen I, Bergstrom G, Ljungquist T, Bodin L (2005). A 3-year follow-up of a multidisciplinary rehabilitation programme for back and neck pain. Pain.

[26] Hees H, De Vries G, Koeter M, Schene A (2012). Adjuvant occupational therapy improves long-term depression recovery and return-to-work in good health in sick-listed employees with major depression: results of a randomised controlled trial. Occup Environ Med.

[27] Hees H, Koeter M, De Vries G, Ooteman W, Ah S (2010). Effectiveness of adjuvant occupational therapy in employees with depression: design of a randomized controlled trial. BMC Public Health.

[28] Vlasveld M, Anema J, Beekman A, Van Mechelen W, Hoedeman R, Van Marwijk H (2008). Multidisciplinary collaborative care for depressive disorder in the occupational health setting: design of a randomised controlled trial and cost-effectiveness study. BMC Health Serv Res.

[29] Vlasveld M, Van Der Feltz-Cornelis C, Adre H, Anema J, Hoedeman R, Van Mechelen W (2012). Collaborative care for sick-listed workers with major depressive disorder: a randomised controlled trial from the Netherlands Depression Initiative aimed at return to work and depressive symptoms. Occup Environ Med.

[30] Loisel P, Abehaim L, Durand P, Esdaile J, Suissa S, Gosselin L (1997). A population-based, randomized clinical trial on back pain management. Spine.

[31] Loisel P, Gosselin L, Durand P, Lemaire J, Poitras S, Abehaim L (2001). Implementation of a participatory ergonomics program in the rehabilitation of workers suffering from subacute back pain. Appl Ergon.

[32] Loisel P, Lemaire J, Poitras S, Durand M-J, Champagne F, Stock S (2002). Cost-benefit and cost-effectiveness analysis of a disability prevention model for back pain management: a six year follow up study. Occup Environ Med.

[33] Noordik E, Van Der Klink JJ, Geskus RB, De Boer MR, Van Dijk FJ, Nieuwenhuijsen K (2013). Effectiveness of an exposure-based return-to-work program for workers on sick leave due to common mental disorders: a cluster-randomized controlled trial. Scand J Work Environ Health.

[34] Noordik E, Van Dijk F, Nieuwenhuijsen K, Van Der Klink JL (2009). Effectiveness and cost-effectiveness of an exposure-based return-to-work programme for patients on sick leave due to common mental disorders: design of a cluster-randomized controlled trial. BMC Public Health.

[35] Tamminga SJ, De BoerAGEM, Verbeek JHaM, Taskila T, Frings-Dresen MHW (2010). Enhancing return-to-work in cancer patients, development of an intervention and design of a randomized controlled trial. BMC Cancer.

[36] Tamminga S, Verbeek JHaM, Bos M, Fons G, Kitzen JJEM, Plaisier PW (2013). Effectiveness of a hospital-based work support intervention for female cancer patients—a multi-centre randomised controlled trial. PLoS One.

[37] Spelten ER, Sprangers MA, Verbeek J (2002). Factors reported to influence the return to work of cancer survivors: a literature review. Psychooncology.

[38] Feuerstein M, Huang GD, Ortiz J, Shaw WS, Miller V, Wood P (2003). Integrated case management for work-related upper-extremity disorders: impact of patient satisfaction on health and work status. J Occup Environ Med.

[39] Carroll C, Rick J, Pilgrim H, Cameron J, Hillage J (2010). Workplace involvement improves return to work rates among employees with back pain on long-term sick leave: a systematic review of the effectiveness and cost-effectiveness of interventions. Disabil Rehabil.

[40] Furlan AD, Gnam WH, Carnide N, Irvin E, Amick Iii BC, Derango K (2012). Systematic review of intervention practices for depression in the workplace. J Occup Rehabil.

[41] Odeen M, Magnussen LH, Maeland S, Larun L, Eriksen HR, Tveito TH (2013). Systematic review of active workplace interventions to reduce sickness absence. Occup Med.

[42] Palmer K, Harris E, Linaker C, Al E (2012). Effectiveness of community and workplace-based interventions to manage musculoskeletal-related sickness absence and job loss: a systematic review. Rheumatology.

[43] Pomaki G, Franche R, Murray E, Khushrushahi H, Lampinen TM (2012). Workplace-based work disability prevention interventions for workers with common mental health conditions: a review of the literature. J Occup Rehabil.

[44] Schandelmaier S, Ebrahim S, Burkhardt SCA, De Boer AGEM, Zumbrunn T, Guyatt GH (2012). Return to work coordination programmes for work disability: a meta-analysis of randomised controlled trials. PLoS One.

[45] Nevala N, Pehkonen I, Koskela I, Ruusuvuori J, Anttilla H (2015). Workplace accommodation among persons with disabilities: a systematic review of its effectiveness and barriers or facilitators. J Occup Rehabil.

[46] American College of Occupational and Environmental Medicine. Guidelines for preventing needless work disability by helping people stay employed. American College of Occupational and Environmental Medicine; 2006. 10.1097/01.jom.0000235915.61746.0d 16966965

[47] Watson Wyatt Worldwide. Dashboard for success. How best performers do it. Arlington: Watson Wyatt Worldwide; 2007.

[48] Adya M, Cirka C, Mitchell K (2012). Final report: corporate return to work policies and practices: a national study.

[49] Podniece Z, Pinder A, Yeomans L, Van Den Heuvel S, Blatter B, Verjens M (2007). Work-related muskuloskeletal disorders: back to work report.

[50] International Labour Office (2011). Disability in the workplace: employers’ organizationas and business networks.

[51] International Labour Office. Managing disability in the workplace. ILO code of practice. Geneva: International Labour Office; 2002.

[52] Life Insurance Company of North America (Cigna). Employer’s guide to creating a successful return-to-work (RTW) program. Bloomfield: Life Insurance Company of North America (CIGNA); 2009.

[53] Bunn Iii WB, Baver RS, Ehni TK, Stowers AD, Taylor DD, Holloway AM (2006). Impact of a muskuloskeletal disability management program on medical costs and productivity in a large manufacturing companyh. The American Journal of Managed Care..

[54] Wynn R, Mcananey D (2004). Employment and disability: back to work strategies.

[55] Zeitzer I, Johnson J (2001). Who returns to work and why: evidence and policy implications from a new disability and work reintegration study.

[56] Wleklinski B, Salon R, Taylor B. Best practices in employee retention and return-to-work: an in-depth look inside an exemplary american corporation. Washington, DC: National Disability Institute’s LEAD Center: Leadership for the Employment and Economic Advancement of People with Disabilities; 2014.

[57] Mitchell K. The return to work dividend: protecting productivity. Stay-at-work and back-to-work strategies: lessons from the private sector. Washington, DC: US Senate Committee on Health, Education, Labor and Pensions; 2012.

[58] Disability Management Employer Coalition (2011). Best practices in return to work.

[59] American College of Occupational and Environmental Medicine (2006). Preventing needless work disability by helping people stay employed. J Occup Environ Med.

[60] Workers’ Compensation Board of Nova Scotia (2015). Return-to-work: getting started.

[61] American College of Occupational and Environmental Medicine (2005). Preventing needless work disability by helping people stay employed: a report from the stay-at-work & return-to-work committee of the American College of Occupational & Environmental Medicine.

[62] Bültmann U, Brouwer S, Schultz IZ, Gatchel RJ (2016). Individual-level psychosocial factors and work disability prevention. Handbook of return-to-work: from research to practice.

[63] Shaw W, Kristman Vl, Vezina N. Workplace Issues. In: Patrick Loisel JRA, editor. Handbook of work disability. New York: Springer; 2013. p. 163–82.

[64] Kraaijeveld R, Schaafsma F, Boot C, Shaw W, Bültmann U (2013). Implementation of the participatory approach to increase supervisors’ self-efficacy in supporting employees at risk for sick leave; design of a randomised controlled trial. BMC Public Health.

[65] British Columbia (Public Service Employee Relations Commission) v. BCGSEU. Supreme court reporter: Supreme Court of Canada; 1999.

[66] Hall R. Light duty, limited duty or modified duty assignments: What the ADA and workers’ comp require. Workforce magazine. 2000. Accessed 2 Dec 2015.

[67] Lambeek L, Bosmand J, Van Royen B, Van Tulder M, Van Mechelen W, Anema J (2010). Effect of integrated care for sick listed patients with chronic low back pain: economic evaluation alongside a randomised controlled trial. Br Med J.

[68] Lambeek L, Van Mechelen W, Buijs P, Loisel P, Anema J (2009). An integrated care program to prevent work disability due to chronic low back pain: a process evaluation within a randomized controlled trial. BMC Musculoskelet Disord.

[69] Van Oostrom S, Van Mechelen W, Terluin B, De Vet H, Anema J (2009). A participatory workplace intervention for employees with distress and lost time: a feasibility evaluation within a randomized controlled trial. J Occup Rehabil.

[70] Anderson J, Douma, F. Telework for workers with disabilities pilot projects synthesis report. In: Office of Disability Employment Policy USDoL, editor. Minneapolis; 2009.

[71] Williams-Whitt K (2007). Impediments to disability accommodation. Relat Ind.

[72] Tveito T, Sembajwe G, Boden L, Dennerlein J, Wagner G, Kenwood C (2014). Impact of organizational policies and practices on workplace injuries in a hospital setting. J Occup Environ Med.

[73] Amick Iii BC, Habeck RV, Hunt A, Fossle AH, Chapin A, Keller RB (2000). Measuring the impact of organizational behaviors on work disability prevention and management. J Occup Rehabil.

[74] Pagell M, Dibrell C, Veltri A, Maxwell E (2014). Is an efficacious operation a safe operation: the role of operational practices in worker safety outcomes. IEEE Trans Eng Manag.

[75] Schein EH (2010). Organizational culture and leadership.

[76] Woodman R (2014). The role of internal validity in evaluation research on organizational change interventions. J Appl Behav Sci.

[77] Seligman M, Csikszentmihalyi M (2000). Positive psychology: an introduction. Am Psychol.

[78] Sheldon K, Kashdan T, Steger M (2011). Designing positive psychology: taking stock and moving forward.

[79] Driessen M, Proper K, Anema J, Knol D, Bongers P, Van Der Beek A (2010). Participatory ergonomics to reduce exposure to psychosocial and physical risk factors for low back pain and neck pain: results of a cluster randomised controlled trial. Occup Environ Med.

[80] Driessen M, Proper K, Anema J, Knol D, Bongers P, Van Der Beek A (2011). The effectiveness of participatory ergonomics to prevent low-back and neck pain—results of a cluster randomized controlled trial. Scand J Work Environ Health.

[81] Driessen M, Proper K, Van Tulder M, Anema J, Bongers P, Van Der Beek A (2010). The effectiveness of physical and organisational ergonomic interventions on low back pain and neck pain: a systematic review. Occup Environ Med.

[82] Nielsen K, Abildgaard J (2013). Organizational interventions: a research-based framework for the evaluation of both process and effects. Work Stress Int J Work Health Organ.

[83] Nielsen K (2013). Review article: How can we make organizational interventions work? Employees and line managers as actively crafting interventions. Hum Relat.

[84] Barends E, Janssen B, Ten Have W, Ten Have W (2014). Effects of change interventions: What kind of evidence do we really have?. J Appl Behav Sci.

[85] Armenakis A, Bedeian A (1999). Organizational change: a review of theory and research in the 1990s. J Manag.

[86] Amis J, Aissaoui R (2013). Readiness for change: an institutional perspective. J Change Manag.

[87] Vakola M (2013). Multilevel readiness to organizational change: a conceptual approach. J Change Manag.

[88] Nicholas MK, Main CJ, Shaw WS, Tetrick LE, Ehrhart MG, Pransky G. Implementation science and employer disability practices: Should implementation factors be imbedded in research designs? J Occup Rehabil. 2016. 10.1007/s10926-016-9677-7 PMC5104783 27796914

