# Supplementary material for: Workplace Interventions to Prevent Disability from Both the Scientific and Practice Perspectives: A Comparison of Scientific Literature, Grey Literature and Stakeholder Observations
Source: J Occup Rehabil. 2016 Sep 10;26(4):417–33. doi: 10.1007/s10926-016-9664-z (PMC5104758; doi:10.1007/s10926-016-9664-z)
Supplement: Supplementary file 1 — Supplementary material 1 (DOCX 35 kb) [file 10926_2016_9664_MOESM1_ESM.docx]

**Appendix A**

| **Cochrane Review Study Descriptions** | |
| --- | --- |
| Anema [1,2] | RCT in two steps. Sicklisted with LBP 2 to 6 weeks. Step 1: Workplace intervention (workplace assessment, work modifications, case management, involvment of all stakeholders, n = 96) vs. usual care (n = 100). Step 2: Participants sick listed at 8 weeks randomised to graded activity (n = 55) vs. usual care (n = 57). |
| van Oostrom [3-5] | RCT. Sick listed with distress for 2 to 8 weeks. Participatory workplace intervention (RTW plan developed with assigned RTW coordinator. 3 meetings: 1. employee and RTW c to identify obstacles for RTW; 2: supervisor and RTW c to identify obstacles for RTW; 3: all 3 parties to work out solutions. n = 73) vs. usual care (n = 72). |
| Arnetz [6] | RCT. Workers sick listed with musculoskeletal disorders (MSD). Proactive RTW management (coordinator with a more proactive role assigned tosick listed workers, involving all stakeholders to meet and work out a solution, n = 65) vs. usual case management (n = 72). |
| Blonk [7] | RCT . Self-employed workers sick listed with psychological complaints. CBT (11 45-min. sessisons, every other week, psychologist, n = 40) vs. combined interention (5 to 6 1-hour sessions twice a week, labour expert delivering CBT-based stress management training, n = 40) vs. usual care (n = 42). |
| Bultmann [8] | RCT. Sick listed with MSD 4 to 12 weeks. Coordinated and tailored work rehabilitation (screening for work disability and identification of barriers, then formulation of a RTW plan, n = 68) vs. usual care (n = 51). |
| Lambeek [9-11] | Compare LBP interventions: 1) a multidisciplinary workplace intervention with particiaptory ergonomics, graded activity using CBT principles, and 2) usual care with a medical specialist, occupational physician, GP and allied health professionals |
| Busch & Jensen [12,13] | Compare back pain interventions: 1) behavior-oriented physiotherapy, 2) cognitive-behavioral therapy, 3) usual care |
| Hees [14,15] | Compare major depressive disorder interventions (MDD) for RTW: 1) occupational intervention, and 2) usual care |
| Vlasveld [16,17] | Compare MDD interventions for RTW: 1) collaborative care, 2) usual care |
| Loisel [18-20] | Occupational and ergonomic intervention, participatory ergonomics compared to clinical intervention for **sub-acute back pain** |
| Noordik [21,22] | Exposure-based return-to-work (RTW) intervention compared to guideline-directed care-as-usual (CAU) by occupational physicians among workers with **common mental disorders** |
| Tamminga [23,24] | Intervention involved improvement of communication between treating and occupational physicians. Researchers asked patient’s occupational physician to organise a meeting with the patient and the supervisor for return-to-work plan compared to usual care for **female cancer patients**. |
| Verbeek [25] | Early management of workers with **low back pain** by occupational physicians, as compared with management by the supervisor only. |
| Feuerstein [26] | Integrated case management (ergonomic and problem-solving intervention) to **work-related upper-extremity disorders** was examined in relation to patient satisfaction, future symptom severity, function, and RTW. Workers with work-related upper-extremity disorder (n 205) were randomly assigned to usual care or ICM intervention. |

| **Non-Cochrane Systematic Review Descriptions** | |
| --- | --- |
| Carroll, 2010 | " In one group of four trials, the intervention consisted of meetings among an occupational health practitioner, employee and employer [30], or agreed work modifications following meetings among the employee, employer and occupational health practitioners [19–21] A second group of three trials focused on exercise therapy delivered either at the workplace [22,28] or developed after a workplace visit and with the employee’s workplace in mind [24,25]. In the final two trials, one intervention combined a degree of employer consultation (communication between occupational health practitioner and the employer) with possible exercise, workplace modification and usual care [26], whereas the second combined cognitive behavioral therapy (CBT) and exercise [23]." |
| Furlan, 2012 | To identify evidence-based programs, or intervention approaches that could be implemented or facilitated by employers to manage workers’ depression and reduce associated productivity losses. |
| Gensby, 2013 | WDPM programs that are managed/implemented at the workplace, involving key workplace players, addressing all types of work disability, and with a clear link between intervention and the program provided. |
| Odeen, 2013 | RCTs of active interventions with at least some part of the intervention at the workplace or at the initiative of the employer. The intervention had to be aimed at one or several specific workplaces and at behavioral change. Interventions recruiting participants from clinical settings or economic claim databases were excluded unless recruitment was done at a predefined set of workplaces. |
| Palmer, 2012 | At the workplace level, approaches included: ergonomic modifications (e.g. lighter duties, reduced hours); and interventions directed at managers (education and advice).and/or psychosocial risk assessments—aimed at the individual (e.g. how well an exercise programme matched job demands) or at identifying and controlling workplace risks (participatory ergonomic assessments,involving the individual, his/her manager and union); ergonomic changes to the physical environment; job |
| Pomaki, 2012 | Workplace-based work disability prevention interventions for workers with common mental health conditions. Three main intervention elements identified across the studies: 1. Facilitation of access to clinical treatment outside the workplace; 2. Workplace-based psychological intervention; 3. Facilitation of navigation through the disability management system. |
| Schandelmaier, 2012 | Return to work coordination programmes for work disability across diagnoses. 9 interventions included, 8 for workers with musculoskeletal and 1 for workers with mental complaints. RCTs. Compared to usual practise. Employer initiated RTW coordination programmes excluded because of focus on prevention and easier access to implement changes in the workplace. |
| Nevala, 2015 | Work accommodation across disability types |

| **Grey Literature Document Descriptions** | |
| --- | --- |
| Article 1 [27] | American College of Occupational and Environmental Medicine: Guidelines for employers and other stakeholders in the RTW/SAW process. |
| Article 12 [28] | Report on actions and trends in health care benefit programs - uses cost to employer as the benchmark, does not evaluate health or other outcomes |
| Article 13 [29] | Summary of Franche et al (2005) literature review of RTW interventions |
| Article 14 [30] | National study of corporate RTW policies and practices |
| Article 15 [31] | Review of academic literature on interventions for RTW with MSD |
| Article 16 [32] | 12 case studies of employer organizations and business networks as a tool to improve disability practices in the workplace |
| Article 17 [33] | ILO code providing practical guidance on disability management in the workplace |
| Article 18 [34] | Cigna Guide to RTW program |
| Article 19 [35] | Nova Scotia guide to RTW |
| Article 20 [36] | Staged communication and educational intervention targeting physicians to improve care of MSK conditions and reduce worker absenteeism |
| Article 22 [37] | Report to physicians on SAW/RTW |
| Article 23 [38] | European case studies on general **disability/chronic illness** management. Covers CMD and MSC |
| Article 24 [39] | Work undertaken in mid-1990s on **back problems** in 6 countries |
| Article 25 [40] | A case study on a corporation on disability prevention and management |
| Article 27 [41] | Stay-at-Work and Back-to-Work Strategies: Lessons from the Private Sector |
| Article 29 [42] | A panel of HR , disability, health and productivity managers, from a variety of companies explored the components of effective RTW programs |

**References for Appendix**

1. Anema J, Steenstra I, Urlings I, Bongers P, De Vroome EMM, Van Mechelen W. Participatory ergonomics as a return-to-work intervention: a future challenge? Am J Ind Med. 2003;44:273-81.

2. Anema J, Steenstra I, Bongers P, De Vet H, Knol D, Loisel P. Multidisciplinary rehabilitation for subacute low back pain: Graded activity or workplace intervention or both? A randomized controlled trial. Spine (Phila Pa 1976). 2007;32:291-8.

3. Van Oostrom S, Anema J, Terluin B, De Vet H, Van Tulder M, Van Mechelen W. Cost-effectiveness of a workplace intervention for sick-listed employees with common mental disorders: design of a randomized controlled trial. BMC public health. 2008;8:1-9.

4. Van Oostrom S, Heymans M, De Vet H, Van Tulder M, Van Mechelen W, Anema J. Economic evaluation of a workplace intervention for sick-listed employees with distress. Occup Environ Med. 2010;667:603-10.

5. Van Oostrom S, Van Mechelen W, Terluin B, De Vet H, Knol D, Anema J. A workplace intervention for sick-listed employees with distress: results of a randomised controlled trial. Occup Environ Med. 2010;67:596-602.

6. Arnetz B, Sjogren B, Rydehn B, Meisel R. Early workplace intervention for employees with musculoskeletal-related absenteeism: a prospective controlled intervention study. J Occup Environ Med. 2003;45:499-506.

7. Blonk R, Brenninkmeijer V, Lagerveld S, Houtman I. Return to work: A comparison of two cognitive behavioural interventions in cases of work-related psychological complaints among the self-employed. Work Stress. 2006;20:129-44.

8. Bultmann U, Shersen D, Olsen J, Hansen C, Lund T, Kilsgaard J. Coordinated and tailored work rehabilitation: A randomized controlled trial with economic evaluation undertaken with workers on sick leave due to musculoskeletal disorders. J Occup Rehabil. 2009;19:81-93.

9. Lambeek L, Anema J, Van Royen B, Buijs P, Wuisman P, Van Tulder M. Multidisciplinary outpatient care program for patients with chronic low back pain: design of a randomized controlled trial and cost-effectiveness study. BMC public health. 2007;7:1-11.

10. Lambeek L, Bosmand J, Van Royen B, Van Tulder M, Van Mechelen W, Anema J. Effect of integrated care for sick listed patients with chronic low back pain: economic evaluation alongside a randomised controlled trial. Br Med J. 2010;341:c6414.

11. Lambeek L, Van Mechelen W, Knol D, Loisel P, Anema J. Randomised controlled trial of integrated care to reduce disability from chronic low back pain in working and private life. Br Med J. 2010;340:c1035.

12. Busch H, Bodin L, Bergstrom G, Jensen I. Patterns of sickness absence a decade after pain-related multidisciplinary rehabilitation. Pain. 2011;152:1727-33.

13. Jensen I, Bergstrom G, Ljungquist T, Bodin L. A 3-year follow-up of a multidisciplinary rehabilitation programme for back and neck pain. Pain. 2005;115:273-83.

14. Hees H, De Vries G, Koeter M, Schene A. Adjuvant occupational therapy improves long-term depression recovery and return-to-work in good health in sick-listed employees with major depression: results of a randomised controlled trial. Occup Environ Med. 2012;70:525-60.

15. Hees H, Koeter M, De Vries G, Ooteman W, Ah S. Effectiveness of adjuvant occupational therapy in employees with depression: design of a randomized controlled trial. BMC public health. 2010;10:1-9.

16. Vlasveld M, Anema J, Beekman A, Van Mechelen W, Hoedeman R, Van Marwijk H. Multidisciplinary collaborative care for depressive disorder in the occupational health setting: design of a randomised controlled trial and cost-effectiveness study. BMC Health Serv Res. 2008;8:1-12.

17. Vlasveld M, Van Der Feltz-Cornelis C, Adre H, Anema J, Hoedeman R, Van Mechelen W. Collaborative care for sick-listed workers with major depressive disorder: a randomised controlled trial from the Netherlands Depression Initiative aimed at return to work and depressive symptoms. Occup Environ Med. 2012;70:223-30.

18. Loisel P, Abehaim L, Durand P, Esdaile J, Suissa S, Gosselin L. A population-based, randomized clinical trial on back pain management. Spine (Phila Pa 1976). 1997;22:2911-8.

19. Loisel P, Gosselin L, Durand P, Lemaire J, Poitras S, Abehaim L. Implementation of a participatory ergonomics program in the rehabilitation of workers suffering from subacute back pain. Appl Ergon. 2001;32:53-60.

20. Loisel P, Lemaire J, Poitras S, Durand M-J, Champagne F, Stock S. Cost-benefit and cost-effectiveness analysis of a disability prevention model for back pain management: a six year follow up study. Occup Environ Med. 2002;59:807-15.

21. Noordik E, Van Der Klink JJ, Geskus RB, De Boer MR, Van Dijk FJ, Nieuwenhuijsen K. Effectiveness of an exposure-based return-to-work program for workers on sick leave due to common mental disorders: a cluster-randomized controlled trial. Scand J Work Environ Health. 2013;39:144-54. doi:10.5271/sjweh.3320.

22. Noordik E, Van Dijk F, Nieuwenhuijsen K, Van Der Klink JL. Effectiveness and cost-effectiveness of an exposure-based return-to-work programme for patients on sick leave due to common mental disorders: design of a cluster-randomized controlled trial. BMC public health. 2009;9:1-11.

23. Tamminga S, Verbeek JHaM, Bos M, Fons G, Kitzen JJEM, Plaisier PW. Effectiveness of a hospital-based work support intervention for female cancer patients - a multi-centre randomised controlled trial. PLoS One. 2013;8:1-9.

24. Tamminga SJ, De Boer AGEM, Verbeek JHaM, Taskila T, Frings-Dresen MHW. Enhancing return-to-work in cancer patients, development of an intervention and design of a randomized controlled trial. BMC Cancer. 2010;10:345-54.

25. Spelten ER, Sprangers MA, Verbeek J. Factors reported to influence the return to work of cancer survivors: a literature review. Psychooncology. 2002;11:124-31.

26. Feuerstein M, Huang GD, Ortiz J, Shaw WS, Miller V, Wood P. Integrated case management for work-related upper-extremity disorders: impact of patient satisfaction on health and work status. J Occup Environ Med. 2003;45:803-12.

27. American College of Occupational and Environmental Medicine. Guidelines for Preventing Needless Work Disability by Helping People Stay Employed. American College of Occupational and Environmental Medicine, 2006.

28. Watson Wyatt Worldwide. Dashboard for success. How best performers do it. Arlington, VA: Watson Wyatt Worldwide, 2007.

29. Institute for Work & Health. Seven 'Principles' for Successful Return to Work. <https://www.iwh.on.ca/seven-principles-for-rtw:> Institute for Work & Health,2014.

30. Adya M, Cirka C, Mitchell K. Final report: Corporate return to work policies and practices: A national study. Syracuse, NY: Burton-Blatt Institute, Syracuse University, 2012.

31. Podniece Z, Pinder A, Yeomans L, Van Den Heuvel S, Blatter B, Verjens M et al. Work-related muskuloskeletal disorders: Back to work report. Brussels, Belgium: European Agency for Safety and Health at Work, 2007.

32. International Labour Office. Disability in the workplace: Employers' organizationas and business networks. Geneva, Switzerland: International Labour Office, 2011.

33. International Labour Office. Managing disability in the workplace. ILO code of practice. Geneva, Switzerland: International Labour Office, 2002.

34. Life Insurance Company of North America (Cigna). Employer's guide to creating a successful return-to-work (RTW) program. Bloomfield, CT: Life Insurance Company of North America (CIGNA), 2009.

35. Anderson J, Douma, F. Telework for workers with disabilities pilot projects synthesis report. In: Office of Disability Employment Policy USDoL, editor. Minneapolis, MN2009.

36. Bunn Iii WB, Baver RS, Ehni TK, Stowers AD, Taylor DD, Holloway AM et al. Impact of a muskuloskeletal disability management program on medical costs and productivity in a large manufacturing companyh. The American Journal of Managed Care. 2006;12:SP27-32.

37. American College of Occupational and Environmental Medicine. Preventing needless work disability by helping people stay employed: A report from the stay-at-work & return-to-work committee of the American College of Occupational & Environmental Medicine. Elk Grove Village, IL: American College of Occupational & Environmental Medicine, 2005.

38. Wynn R, Mcananey D. Employment and disability: Back to work strategies. Copenhagen, Denmark: European Foundation for the Improvement of Living and Working Conditions, 2004.

39. Zeitzer I, Johnson J. Who returns to work and why: Evidence and policy implications from a new disability and work reintegration study. Geneva, Switzerland: International Social Security Association Research Programme, 2001.

40. Wleklinski B, Salon R, Taylor B. Best Practices in Employee Retention and Return-to-Work: An In-Depth Look Inside an Exemplary American Corporation. Washington, D.C.: National Disability Institute's LEAD Center: Leadership for the Employment and Economic Advancement of People with Disabilities, 2014.

41. Mitchell K. The return to work dividend: Protecting productivity. Stay-at-work and back-to-work strategies: Lessons from the private sector. Washington, D.C.: US Senate Committee on Health, Education, Labor and Pensions, 2012.

42. Disability Management Employer Coalition. Best practices in return to work. San Diego, CA: Disability Management Employer Coalition, 2011.
